# Relative validity of an intelligent ordering system to estimate dietary intake among university students from a medical school in Shanghai, China

**DOI:** 10.1186/s12966-024-01619-1

**Published:** 2024-07-04

**Authors:** Yimeng Zhang, Dantong Gu, Mengyun Luo, Shaojie Liu, Hong Peng, Yingnan Jia

**Affiliations:** 1 School of Public Health, Key Lab of Public Health Safety of the Ministry of Education, Fudan University, 130 Dongan Road, Shanghai, 200032 People’s Republic of China; 2 Institute of Otolaryngology, Clinical Research Center, Eye and ENT Hospital, Fudan University, Shanghai, 200031 People’s Republic of China; 3 Prevention Research Collaboration, Sydney School of Public Health, The University of Sydney, Sydney, NSW Australia; 4 Charles Perkins Centre, The University of Sydney, Sydney, NSW Australia; 5 Department of Nutrition, the First Affiliated Hospital of Xiamen University, Xiamen, 361003 China; 6 Health Communication Institute, Fudan University, Shanghai, 200032 China

**Keywords:** Dietary assessment, Relative validity, Intelligent ordering system, Food diary, Medical students

## Abstract

**Background:**

Dietary assessment methods have limitations in capturing real-time eating behaviour accurately. Equipped with automated dietary-data-collection capabilities, the “intelligent ordering system” (IOS) has potential applicability in obtaining long-term consecutive, relatively detailed on-campus dietary records among university students with little resource consumption. We investigated (1) the relative validity of IOS-derived nutrient/food intakes compared to those from the 7-day food diary (7DFD); (2) whether including a supplemental food frequency questionnaire (SFFQ) improves IOS accuracy; and (3) sex differences in IOS dietary intake estimation.

**Methods:**

Medical students (*n* = 221; age = 22.2 ± 2.4 years; 38.5% male and 61.5% female) completed the 7DFD and SFFQ. During the consecutive 7-day survey period, students weighed and photographed each meal before and after consumption. Then, students reviewed their 3-month diet and completed the SFFQ, which includes eight underprovided school-canteen food items (e.g., dairy, fruits, nuts). Meanwhile, 9385 IOS dietary data entries were collected. We used Spearman coefficients and linear regression models to estimate the associations among the different dietary intake assessment methods. Individual- and group-level agreement was assessed using the Wilcoxon signed-rank test, cross-classification, and Bland‒Altman analysis.

**Results:**

IOS mean daily energy, protein, fat, and carbohydrate intake estimations were significantly lower (-15-20%) than those of the 7DFD. The correlation coefficients varied from 0.52 (for added sugar) to 0.88 (for soybeans and nuts), with fruits (0.37) and dairy products (0.29) showing weaker correlations. Sixty-two (milk and dairy products) to 97% (soybeans and nuts) of participants were classified into the same or adjacent dietary intake distribution quartile using both methods. The energy and macronutrient intake differences between the IOS + SFFQ and 7DFD groups decreased substantially. The separate fruit intake measurements from each assessment method did not significantly differ from each other (*p* > 0.05). IOS and IOS + SFFQ regression models generally yielded higher R^2^ values for males than for females.

**Conclusion:**

Despite estimation differences, the IOS can be reliable for medical student dietary habit assessment. The SFFQ is useful for measuring consumption of foods that are typically unavailable in school cafeterias, improving the overall dietary evaluation accuracy. The IOS assessment was more accurate for males than for females.

**Supplementary Information:**

The online version contains supplementary material available at 10.1186/s12966-024-01619-1.

## Introduction

An unbalanced diet is an important preventable risk factor for noncommunicable diseases (NCDs) [1]. In the past three decades, China has experienced a rapid nutrition transition, leaning towards a Westernized diet dominated by increased consumption of high-calorie, animal-sourced, and processed foods, resulting in prevalent NCDs [2–4]. University students are especially vulnerable to unhealthy, highly energy-dense diets attributed to sudden changes in their social networks as well as the absence of parental oversight after leaving home [5]. More importantly, dietary patterns and behaviours developed during university years can exert lasting health effects in later adulthood, such as influencing the risk of obesity and type-2 diabetes [6, 7].

In contrast to many other countries in the world, in China, on-campus canteens are partially funded by the government, which provides convenient and affordable food options; therefore, they are predominantly the best place for dining for university students [8]. According to data from the China Food Consumption Survey, more than half of university students dine at the canteen [9]. This makes it feasible to accurately assess university students’ on-campus food intake, understand their dietary patterns, and identify existing nutritional issues to inform future intervention strategies in China.

The literature has explored the nutritional intake and dietary habits of university students [10–12]. However, commonly used measurement methods, such as 24-hour recalls and food frequency questionnaires, are labour-intensive, time consuming, and prone to reporting bias [13]. Researchers in epidemiology and nutrition have increasingly explored the integration of emerging technologies to develop more advanced dietary assessment instruments, including image-based assessments [14], smartphone apps [15], and wearable sensors [16]. These new techniques can, to a certain extent, provide real-time data collection and improve data quality. However, most of these methods largely rely on participant compliance or emerging technologies that are still being researched and developed [17]. Previous studies have suggested that automated electronic sales data can be used as a promising alternative measurement for population dietary surveillance [18, 19]. Nevertheless, the challenges of data availability and nutrient data linkage have resulted in some studies presenting their research findings using dietary behaviour indicators [20] or diet-quality indices [21, 22] without providing absolute estimates of dietary intake. It is also important to note that the food shopping data in these studies usually reflect the shopping habits of a household rather than an individual. Therefore, using such data to infer insights about individual food intake may lead to less precise results [23].

Moreover, Chinese cuisine typically blends various components, thereby modifying its initial appearance. Even with current technology, it can be challenging to discern the makeup of ingredients quickly and accurately in dishes. Therefore, new technologies for assessing dietary intake are not suitable for long-term follow-up studies with large sample sizes. Although sales data could be an appropriate approach, those data do not provide individual purchase information.

To address the issues mentioned above, we utilized data from the “intelligent ordering system” (IOS) at Fudan University, a system that enables the automatic collection of food ordering data, offering a practical approach for detailed and ongoing dietary assessment among university students. This study aimed to examine the relative validity of the IOS as a substitute approach for assessing on-campus dietary intake in a sample of medical students in Shanghai, China, by comparing it to a self-reported 7-day food diary (7DFD). Given the consistent inadequacy of certain food types in school canteens, such as fresh fruits and dairy products, a brief food frequency questionnaire was designed as an optional supplement to the IOS data, where we tested whether its inclusion could enhance the accuracy of the system in estimating dietary intake. As previous studies have demonstrated differences in dietary intake and eating behaviours between males and females of the same age group [10, 24, 25], we conducted further analysis to explore potential sex disparities in the capacity of the IOS to estimate dietary intake.

## Methods

### Participants and recruitment

**Fig. 1:**
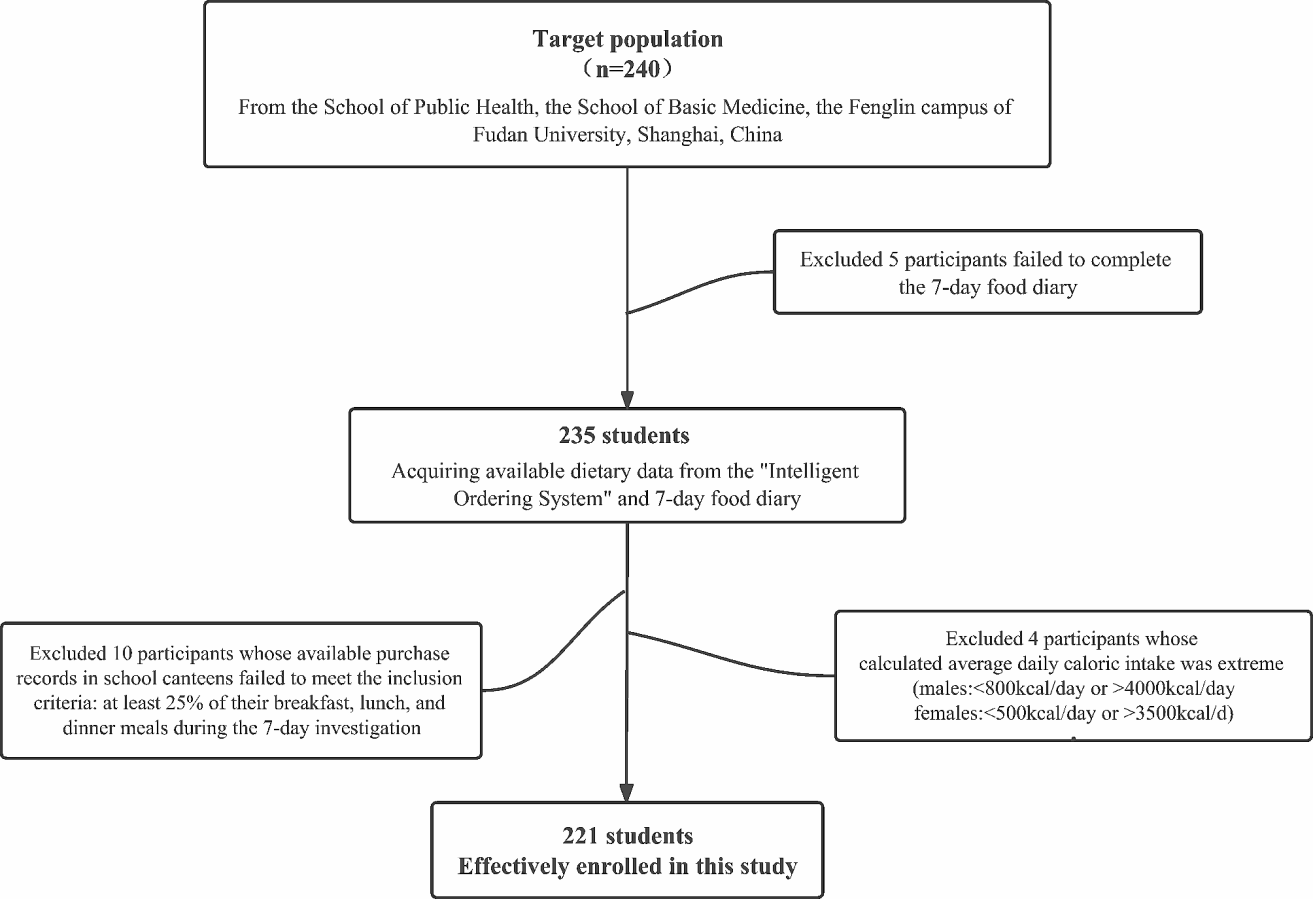
Flow chart of this study

The sample was composed of medical undergraduate and graduate students from the School of Public Health and the School of Basic Medicine at Fenglin Campus, Fudan University, Shanghai, China. Students who resided and studied on other campuses of Fudan University were not considered, as food provisions varied across different campus locations. A convenience sampling strategy was used to recruit students between October and December 2021 through an online advertisement. All eligible participants must be older than 18 at the time of recruitment and have used their identification cards to make purchases at school canteens for at least 25% of their breakfast, lunch, and dinner meals during the investigation period. Based on mealtimes provided by the school canteens, we categorized records before 10:30 a.m. as breakfast, between 10:30 a.m. and 3:30 p.m. as lunch, and after 3:30 p.m. as dinner. Then, we determined the total number of these meals using the 7DFD and integrated these data with IOS records to calculate the proportion of each meal consumed at school canteens. The exclusion criteria included abnormal daily caloric intake [26] (males: <800 kcal/day or > 4000 kcal/day; females: <500 kcal/day or > 3500 kcal/day), desire to gain weight, present use of a therapeutic diet or weight loss diet, and history of eating disorders. Figure 1 displays the flow chart. The final analytic sample included 221 participants (92.08% of the target) with complete dietary data from the IOS, dietary diary, a supplemental food frequency questionnaire, and sociodemographic data. The study procedures were approved by the Ethics Committee of Medical Research, School of Public Health, Fudan University. The study was conducted in accordance with the Declaration of Helsinki, and the protocol was approved by the Ethics Committee of Medical Research, School of Public Health, Fudan University (Project identification code: IRB#2019-01-0726 S). Informed consent was provided by all participants, and they agreed to use their dietary data in the IOS for research purposes.

### Procedures

Participants were recruited in four batches, following the principle of relative balance among sex, major, and grade levels. One week of food consumption was recorded for each participant once, and the four participant batches completed their week of surveys during nonconsecutive weeks between October and December, 2021. Prior to the survey, participants received training on conducting dietary self-assessments and completing the questionnaire. Furthermore, all participants were asked to complete a brief general information questionnaire with questions related to age, sex (male/female), weight, height, household type (resident/nonresident), education (undergraduate/graduate student), major (public health, clinical medicine, or basic medicine), and dietary habits (general diet, Halal diet, or vegetarian diet). Body mass index (BMI) was calculated using self-administered weight and height (kg/m²) and then categorized into 3 mutually exclusive groups according to the national standard for China [27] (underweight: BMI < 18.5 kg/m^2^, normal weight: 18.5–24.0 kg/m^2^, overweight and obesity: BMI > 24.0 kg/m^2^).

The data for the 7DFD and IOS were recorded simultaneously in four one-week batches. Students were strongly encouraged to adhere to their customary dietary routines throughout the study period. When completing the 7DFD, students were instructed to record their recent dietary intake by completing a supplemental food frequency questionnaire (SFFQ).

### Dietary data from the “intelligent ordering system”

The IOS was first officially put in the canteens of the medical school of Fudan University in China in September 2017 [28]. All food purchases in the school canteens are automatically recorded in the IOS. A food database of 1840 dishes was established by weighing the materials before and after cooking. The database includes information on food composition, such as the weight of raw food materials and condiments, as well as the nutrient composition of each meal. Notably, condiments are often used in small quantities, presenting challenges in precise assessments for individuals lacking expertise. Details of the weighing and calculation methods have been described in a previous article [29]. First, raw ingredients and condiments, along with the cooked dish of each recipe, were weighed in the canteen kitchen. Second, servings of each dish were weighed five times at the canteen window, with the average weight in grams used for analysis. Third, the raw food materials of each serving were calculated based on the ratio of the weight in grams of cooked product for each serving. Nutrient composition was determined using the Chinese Food Composition database (version 2019). Researchers were granted access to participants’ dietary data from the IOS, which contained information on meal dates and times, dish names, and the number of servings. Moreover, information on student names and IDs was also collected to link their data from the 7DFD and the SFFQ. Based on the meal data from the IOS and the food composition of each meal during the 7-day survey, we calculated the average intake of each food and condiment per day for study participants dining in the canteens on the Fenglin campus.

### 7-Day food diary (7DFD)

A food diary, also known as a dietary record, is often used as a reference method in validation studies to assess the accuracy of other dietary assessment methods [30]. All participants were provided a printed standardized 7DFD to document their dietary intake, accompanied by a portable electronic scale (Kaifeng Group Co., Ltd. KFJ-A) with a precision of 0.1 g. Guidelines and important considerations were included in the accompanying instructions. On the 7DFD, there were prompts for participants to record details of all meals, snacks, beverages, and nutrient supplements they consumed, including the names of the dishes, time/hour, location, menu ingredients, and weight of each ingredient before and after the meal. When participants dined in the medical school canteens, they were told to report the percentage of dish intake instead of specific portion sizes, as the food composition database of these dishes had been established and constantly updated by the research group. The weight of the cooked ingredients was converted into grams of raw material using specific conversion ratios. For example, the ratio of cooked rice to raw rice is approximately 2, whereas the ratio of cooked meat to raw meat is approximately 0.7. These conversion ratios were determined by weighing outcomes in school canteens. If the nutrition information of some packaged food and bottled drinks was not available in the Chinese Food Composition (version 2019), it was necessary to capture and submit clear images of their nutrition labels.

### Supplemental food frequency questionnaire (SFFQ)

This questionnaire was used to assess the consumption of items that were either unavailable or inadequately provided in the canteen. After completing the 7-day survey, participants were instructed to retrospectively review their dietary habits over the past three months and complete the SFFQ. The SFFQ covered eight food categories: fruits, milk, yogurt, nuts, carbonated beverages, fruit and vegetable juices, other sugar-based beverages, and sugar/chocolate. For each food category, participants were asked about intake frequency and amount per intake. The information on intake frequency (none, less than once a month, 1–3 times a month, 1–3 times a week, 4–6 times a week, once a day, twice a day, or 3 times a day and above) was converted into the number of intakes per day, corresponding to the following values: 0, 0.02, 0.07, 0.29, 0.71, 1, 2, or 3. The daily intake of each food type in grams was calculated based on the reported intake frequency and amount per intake. The data collected from the SFFQ acted as a complement to the IOS. The combined results could provide a more comprehensive record of students’ daily dietary intake.

### Analysis of nutrients and Food groups

The 7DFD was in an open-ended format; thus, the original data were recorded in Excel by a trained researcher and checked by another for accuracy. For composite and mixed dishes dining outside the school canteens, participants generally measured the portion size of the entire dish, which needed to be converted into grams of single food items. To standardize the data, we initially reviewed each food record and generated a list of dishes requiring standardization. Next, two trained dieticians independently created recipes by referring to food pictures taken by study participants, recipes from medical school canteens, or recipes on popular nutrition-related mobile applications. Subsequently, the weight of raw food ingredients in each mixed dish could be calculated based on grams of cooked product and specific conversion ratios. A conference was held within the internal research group with experts to rediscuss the food composition of dishes for which there was an estimated energy difference of over 20% between the two dietitians’ assessments. Finally, the resulting estimates were averaged and combined with on-campus food intake recorded by participants.

The dietary data obtained from the IOS were exported to Excel by the Information Office of Fudan University of Shanghai, China. To calibrate the food intake, we calculated an overall waste rate based on the precise leftover rates reported in the 7DFD. The SFFQ was in a close-ended format, and dietary data were entered using EpiData software. The SFFQ dietary data were converted to the intake (in grams) of each food group per day and then combined with the IOS data to produce adjusted IOS data.

According to the Dietary Guidelines for Chinese Residents (2022) [31], all food materials were converted into ten groups: (1) cereals (whole grains and mixed beans); (2) tubers; (3) vegetables; (4) fruits; (5) animal-sourced foods (meat and poultry, aquatic products, eggs); (6) milk and dairy products; (7) soybeans and nuts; (8) salt; (9) oil; and (10) added sugar (carbohydrate content provided by sugar for cooking, carbonated beverages, other sugar-based beverages, and sugar/chocolate). The average daily nutrient intake was then calculated as total energy (kcal), fat (g), protein (g), and carbohydrate (g). We used the Chinese Food Composition (version 2019) for diet analysis.

### Quality control

As an essential step in assessing dietary intake data, quality control was conducted at different stages of the investigation process. A trial test was conducted to identify potential errors and improve the procedures before the normal investigation. During the data-gathering stage, participants were instructed to take pictures of their food and upload them via email, along with their completed daily food diaries, at the end of each survey day. Several WeChat groups were set up, with each group managed by a research assistant who was responsible for tasks such as collecting photos, reminding participants who failed to upload their daily data on time, answering questions, and providing feedback for any errors. All paper questionnaires were checked for completeness before being collected to minimize missing data. Dietary data were double-entered or cross-checked by a team of researchers who had undergone standardized training. Upon identification of extreme energy intake values, data from the four participants were excluded based on predefined criteria.

### Statistical analysis

Participant characteristics are presented as frequencies and percentages for categorical variables and means and standard deviations (SDs) for continuous variables. The same approach was adopted for the 7DFD, IOS, and IOS + SFFQ datasets.

To evaluate the relative validity of the IOS, nonparametric statistical tests were utilized, including the Wilcoxon signed-rank test to detect significant differences between estimates and Spearman correlation analysis to measure the association between the IOS and the 7DFD. Cross-classification analysis was also conducted to determine the agreement level between the categorization of estimates into four equal parts of the intake distribution, and the percentage of participants classified into the same, same or adjacent, and opposite quartile of intake was calculated. Furthermore, Bland‒Altman analysis [32] was performed to evaluate the bias of the mean differences between the two methods, considering the two methods to be comparable if greater than 95% of the data plots were within the limits of agreement. Linear regression models [33] were used to evaluate the prediction ability of the IOS, using daily energy intake values from the IOS as an independent variable and those of the 7DFD as a dependent variable. In addition, sex stratification was applied in the model. The same analyses were conducted when comparing the IOS + SFFQ and the 7DFD. Statistical significance was established by a two-sided *p* < 0.05. All statistical analyses were conducted in R version 4.1.1 (Auckland, New Zealand) and Python version 3.7 (Wilmington, DE, USA).

## Results

The demographic characteristics of the 221 study participants from the medical school are presented in Table 1. The study participants were 61.5% female, with a mean age of 22.2 ± 2.4 years and an average BMI of 21.3 ± 2.9 kg/m^2^. According to the national BMI cut-off points in China, 158 (71.5%) of the students surveyed were in the normal weight range, and the others were either underweight (15.4%) or classified as overweight or obese (13.1%). The study group was a nearly even mix of undergraduate (53.4%) and higher degree research (46.6%) students, with the majority reporting following a general diet (96.4%) and being urban residents (56.6%).

**Table 1:** The demographic characteristics of study participants (*n* = 221)

Characteristics		Values^a^
Age, Years		22.2 ± 2.4
Gender		
Male		85 (38.5)
Female		136 (61.5)
Education		
Undergraduate		118 (53.4)
Graduate		103 (46.6)
Major		
Public health		111 (50.2)
Clinical medicine or basic medicine		110 (49.8)
Household type		
Urban		125 (56.6)
Countryside		96 (43.4)
Dietary habit^b^		
General diet		213 (96.4)
Halal diet		8 (3.6)
BMI(kg/m^2^)		21.3 ± 2.9
Underweight (< 18.5)		34 (15.4)
Normal weight (18.5–24.0)		158 (71.5)
Overweight and obesity (> 24.0)		29 (13.1)

^a^ Numbers in this table represent means ± standard deviations (SDs) and n (%) for continuous and categorical variables, respectively

^b^ None of our participants followed a vegetarian diet

Table 2 shows the mean daily intake of energy, macronutrients, and food groups as recorded by the IOS and the 7DFD. Overall, the IOS estimated lower mean daily intake for nutrients and food groups than did the 7DFD (*p* < 0.05). The intakes of energy, macronutrients, and most food groups were significantly correlated between the two instruments (r_s_ = 0.70–0.81, *p* < 0.001); however, the correlation coefficients for fruits and milk and dairy products were relatively low (r_s_ = 0.37 and 0.29, respectively, *p* < 0.001). Based on the quartile classification of IOS and 7DFD, more than 79% of the study participants were classified in the same or adjacent quartile for nutrients and other selected food groups, except for fruits (70%) and milk and dairy products (62%) (Table 4).

**Table 2:** Mean difference and correlation coefficient of food intakes between the IOS and the 7DFD (*n* = 221)

	IOS		7DFD		*p*-Value ^a^	Correlation Coefficient ^b^
	Mean ± SD	Median	Mean ± SD	Median		
Energy (kcal)	1668.5 ± 442.0	1607.0	2057.0 ± 501.9	1950.2	< 0.001	0.74*
Protein (g)	67.5 ± 18.8	66.3	84.8 ± 25.9	81.4	< 0.001	0.70*
Fat (g)	67.8 ± 23.7	65.1	82.4 ± 24.9	77.9	< 0.001	0.81*
Carbohydrate (g)	201.9 ± 58.6	198.6	251.8 ± 69.3	241.9	< 0.001	0.75*
Cereals (g)	184.8 ± 63.6	178.1	208.0 ± 72.3	199.0	< 0.001	0.82*
Tubers (g)	31.7 ± 30.8	25.9	38.1 ± 33.3	30.0	< 0.001	0.85*
Vegetables (g)	199.2 ± 102.1	184.5	202.1 ± 102.7	183.8	0.361	0.87*
Fruits (g)	21.2 ± 32.7	5.8	111.2 ± 127.5	72.1	< 0.001	0.37*
Animal-sourced foods (g)	250.3 ± 92.3	241.3	294.5 ± 98.5	282.8	< 0.001	0.82*
Milk and dairy products (g)	20.6 ± 43.1	0.0	89.9 ± 91	72.9	< 0.001	0.29*
Soybeans and nuts (g)	20.3 ± 13	18.3	22.3 ± 14.1	19.9	< 0.001	0.88*
Salt equivalents (g) ^c^	7.2 ± 2.7	6.9	8.0 ± 2.6	7.7	< 0.001	0.78*
Oil (g)	30.5 ± 11.5	30.0	35.1 ± 11.1	34.2	< 0.001	0.79*
Added sugar(g)	10.1 ± 6.0	9.2	20.4 ± 12.3	17.6	< 0.001	0.52*

^a^ Calculated using Wilcoxon signed rank test

^b^ Calculated using Spearman correlation analysis (r_s_). * Significant at the < 0.001 level

^c^ Salt equivalents (g) = sodium (mg)/393

A similar analysis was conducted for the mean daily intake as recorded by the IOS + SFFQ and the 7DFD in Table 3. Considering that the SFFQ only included the intakes of fruits, milk and dairy products, soybeans and nuts, and sugar-sweetened food (added sugar), the comparison results between the IOS + SFFQ and the 7DFD for the other six food groups were consistent with those obtained when comparing the IOS and the 7DFD. The estimated intake of energy and macronutrients was also lower in the IOS + SFFQ group than in the 7DFD group (*p* < 0.001). The IOS + SFFQ overestimated the intake of milk and dairy products, soybeans and nuts, and added sugar (*p* < 0.001). Strong positive correlations were observed between the two dietary assessment methods for most dietary intake, with correlation coefficients exceeding 0.50 (*p* < 0.001); the correlation coefficients for added sugar were relatively low (r_s_ = 0.41, *p* < 0.001). Based on the quartile classification of IOS + SFFQ and 7DFD, it was found that more than 78% of the study participants were classified in the same or adjacent quartile for all dietary intakes (Table 4).

**Table 3:** Mean difference and correlation coefficient of food intakes between the IOS + SFFQ and the 7DFD (*n* = 221)

	IOS + SFFQ		7DFD		*p*-Value ^a^	Correlation Coefficient ^b^
	Mean ± SD	Median	Mean ± SD	Median		
Energy (kcal)	1919.3 ± 481.8	1843.0	2057.0 ± 501.9	1950.2	< 0.001	0.69*
Protein (g)	74.4 ± 19.5	73.6	84.8 ± 25.9	81.4	< 0.001	0.66*
Fat (g)	76.0 ± 26.1	73.1	82.4 ± 24.9	77.9	< 0.001	0.79*
Carbohydrate (g)	241.3 ± 66.9	234.0	251.8 ± 69.3	241.9	< 0.001	0.67*
Cereals (g) ^c^	184.8 ± 63.6	178.1	208.0 ± 72.3	199.0	< 0.001	0.82*
Tubers (g) ^c^	31.7 ± 30.8	25.9	38.1 ± 33.3	30.0	< 0.001	0.85*
Vegetables (g) ^c^	199.2 ± 102.1	184.5	202.1 ± 102.7	183.8	0.361	0.87*
Fruits (g)	114.5 ± 123	80.6	111.2 ± 127.5	72.1	0.559	0.54*
Animal-sourced foods (g) ^c^	250.3 ± 92.3	241.3	294.5 ± 98.5	282.8	< 0.001	0.82*
Milk and dairy products (g)	127.6 ± 121.5	87.0	89.9 ± 91.0	72.9	< 0.001	0.54*
Soybeans and nuts (g)	27.3 ± 21.7	23.5	22.3 ± 14.1	19.9	< 0.001	0.73*
Salt equivalents (g) ^c d^	7.2 ± 2.7	6.9	8.0 ± 2.6	7.7	< 0.001	0.78*
Oil (g) ^c^	30.5 ± 11.5	30.0	35.1 ± 11.1	34.2	< 0.001	0.79*
Added sugar (g)	26.9 ± 28.7	20.0	20.4 ± 12.3	17.6	0.034	0.41*

^a^ Calculated using Wilcoxon signed rank test

^b^ Calculated using Spearman correlation analysis (r_s_). * Significant at the < 0.001 level

^c^ Comparison results between the IOS + SFFQ and the 7DFD were consistent with those obtained when comparing the IOS and the 7DFD, as the SFFQ did not include these food groups

^d^ Salt equivalents (g) = sodium (mg) × 636

**Table 4:** Percentage agreement (%) in quartile distribution of dietary intake between the IOS or IOS + SFFQ and the 7DFD (*n* = 221)

	IOS vs. 7DFD			IOS + SFFQ vs. 7DFD		
	Same quartile	Same or adjacent quartile	Opposite quartile	Same quartile	Same or adjacent quartile	Opposite quartile
Energy (kcal)	54	91	1	50	88	3
Protein (g)	50	91	2	45	90	2
Fat (g)	61	94	0	58	94	1
Carbohydrate (g)	53	88	1	49	86	2
Cereals (g) ^a^	63	97	0	…	…	…
Tubers (g) ^a^	60	92	1	…	…	…
Vegetables (g) ^a^	65	96	0	…	…	…
Fruits (g)	38	70	9	45	84	4
Animal-sourced foods (g) ^a^	59	95	0	…	…	…
Milk and dairy products (g)	35	62	16	44	84	5
Soybeans and nuts (g)	69	97	0	59	92	2
Salt equivalents (g) ^a^	56	91	0	…	…	…
Oil (g) ^a^	55	94	0	…	…	…
Added sugar (g)	42	79	3	43	78	6

^a^ Classification results between the IOS + SFFQ and the 7DFD were consistent with those obtained when comparing the IOS and the 7DFD, thus denoted by “…” to signify repetition

**Fig. 2:**
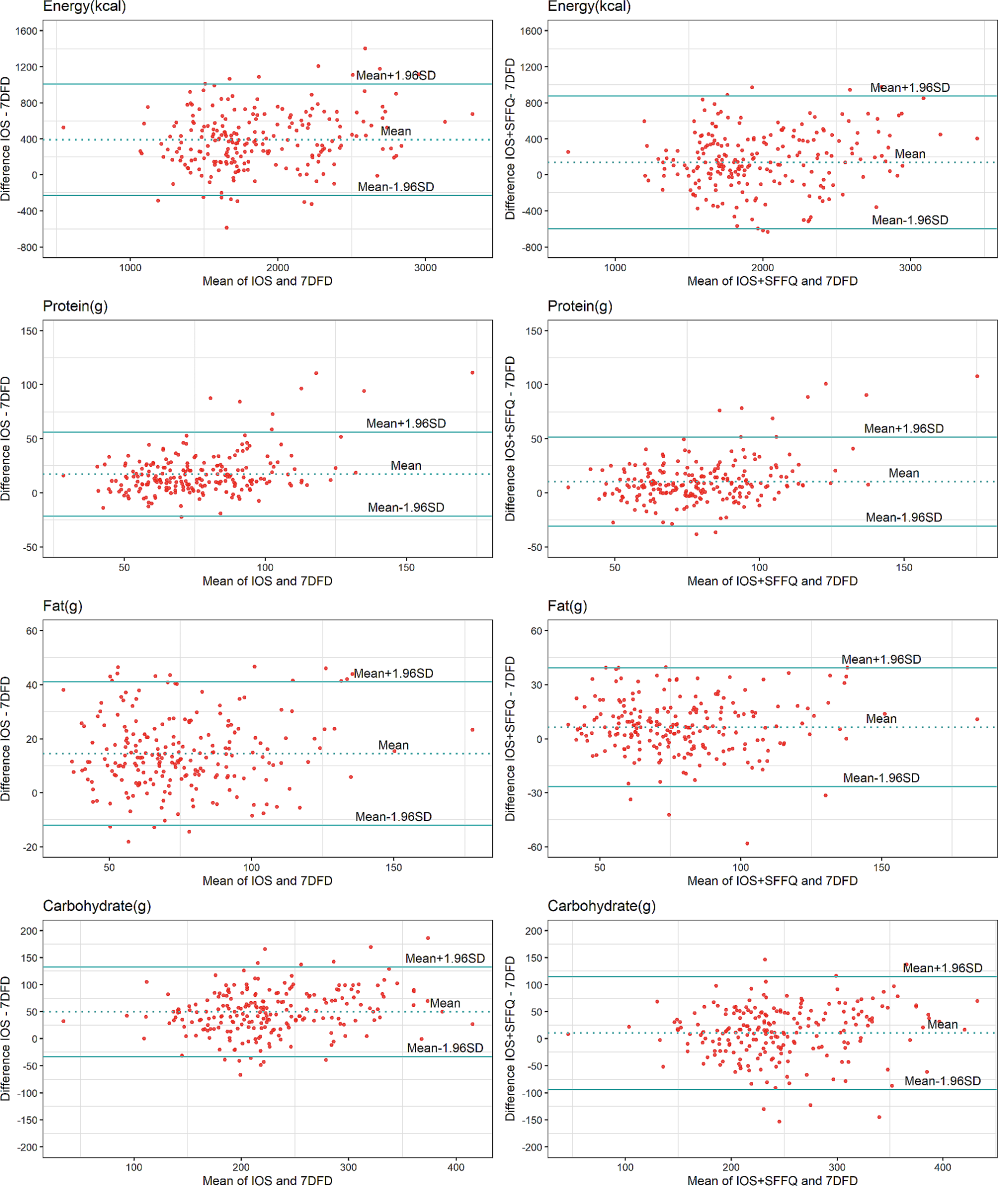
Bland‒Altman plots of agreement between dietary intakes reported in the IOS-7DFD and IOS + SFFQ-7DFD among the study participants (*n* = 221). The dotted line shows the mean difference between the two methods. The solid lines show an agreement interval, representing the mean difference ± 1.96 SD, which covered 95% of the differences between the two methods

Table 5 presents the results from the Bland–Altman analysis and shows the mean difference and 95% LOA for energy, macronutrients, and food groups. The average difference in nutrient intake between the IOS + SFFQ and 7DFD estimates was smaller, which suggested a smaller systematic bias between the two methods. In addition, the limits of agreement were narrower for the chosen nutrients between the IOS and the 7DFD, showing greater agreement between these two methods at the individual level. (Fig. 2)

**Table 5:** Bland-Altman statistics comparing the dietary intake from the IOS or IOS + SFFQ and the 7DFD (*n* = 221)

	IOS vs. 7DFD		IOS + SFFQ vs. 7DFD	
	Mean Difference	95% LOA^a^	Mean Difference	95% LOA^a^
Energy (kcal)	388.5	-230, 1007.1	137.7	-597.8, 873.2
Protein (g)	17.4	-21.4, 56.2	10.4	-30.6, 51.4
Fat (g)	14.5	-12, 41	6.4	-26.5, 39.3
Carbohydrate (g)	49.9	-33, 132.7	10.6	-93.8, 114.9
Cereals (g) ^b^	23.2	-46.2, 91.9	…	…
Tubers (g) ^b^	6.4	-25.6, 38.4	…	…
Vegetables (g) ^b^	2.8	-98.3, 103.1	…	…
Fruits (g)	90.0	-150.4, 330.4	-3.3	-267.7, 261.1
Animal-sourced foods (g) ^b^	44.2	-70, 157.7	…	…
Milk and dairy products (g)	69.3	-101.8, 240.2	-37.7	-264.5, 188.9
Soybeans and nuts (g)	2.01	-10.7, 14.7	-4.99	-43, 33
Salt equivalents (g) ^b^	0.82	-2.5, 4.1	…	…
Oil (g) ^b^	4.58	-8.9, 18	…	…
Added sugar (g)	10.3	-10.4, 30.9	-6.53	-64.5, 51.4

^a^ Lower and upper limit of agreement: mean difference ± 1.96 SD

^b^ Comparison results between the IOS + SFFQ and the 7DFD were consistent with those obtained when comparing the IOS and the 7DFD, thus denoted by “…” to signify repetition

**Fig. 3:**
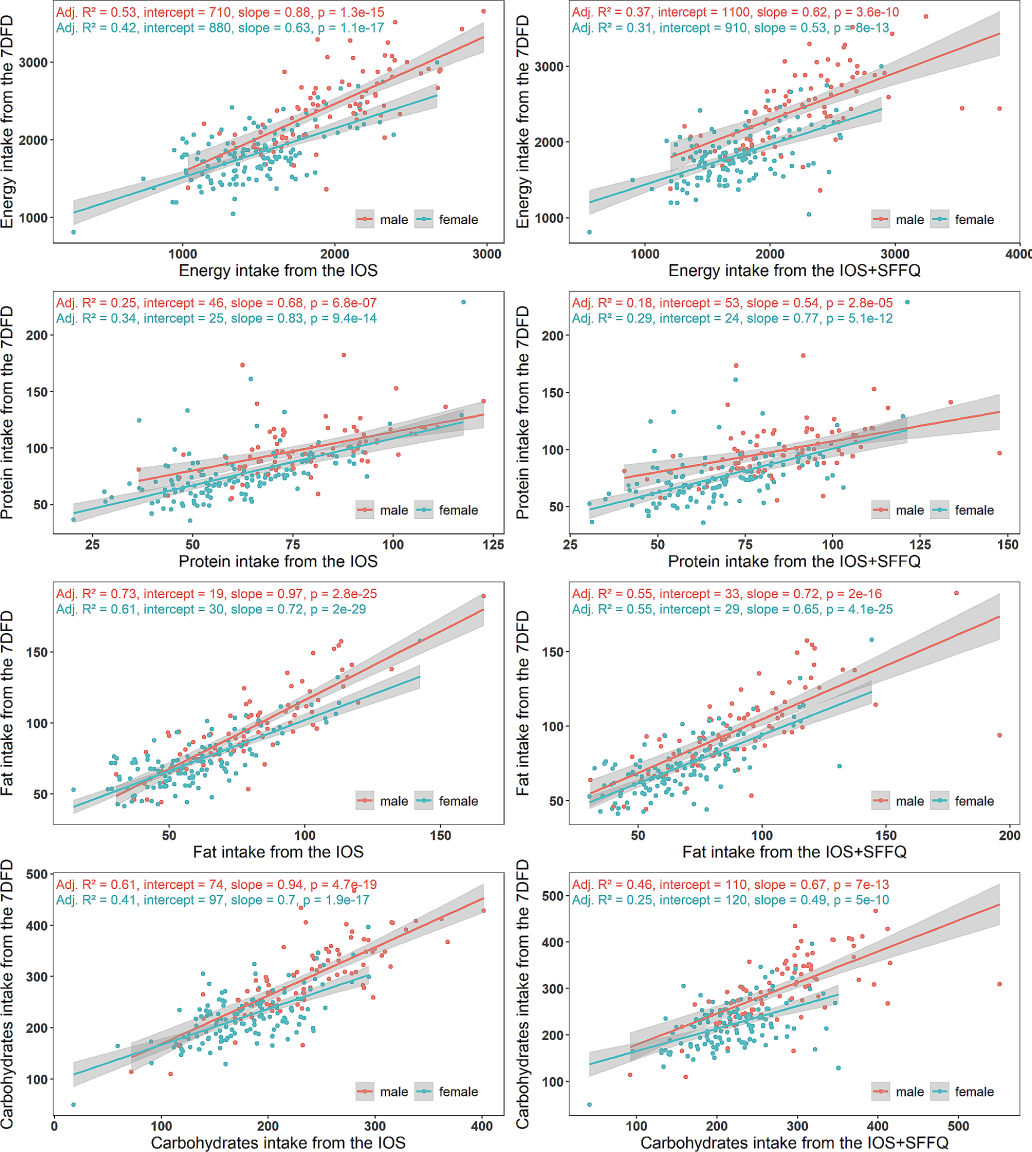
Regression equations predicting nutrient intake using the 7DFD data from reported nutrient intake using two sets of data: IOS and IOS + SFFQ.

Figure 3 displays the predicted models of dietary nutrient (energy, protein, fat, and carbohydrate) intake, which were created to evaluate the association between the actual intake measured by the 7DFD and the reported intake measured by the IOS and IOS + SFFQ. The models were stratified by sex. The coefficient of determination (R^2^) of simple linear regression fitted by the IOS ranged from 0.25 to 0.73. The R^2^ values fitted by the IOS + SFFQ ranged from 0.18 to 0.55. In general, the IOS had a better fitting effect than the IOS + SFFQ in predicting dietary intake for the same nutrients; the predictive performance of using IOS data (with or without the SFFQ) to estimate nutrient intake for males was better than that for females, except for protein.

## Discussion

To our knowledge, this is the first validation study of a canteen ordering system (IOS) to assess dietary intake among university students in China. Our study examined the effectiveness of the IOS in estimating the dietary intake of medical students of campus compared to that of the 7DFD. Furthermore, the relative differences and associations between the data obtained from the IOS and the 7DFD were mostly reasonable and aligned with the literature. This suggests that IOS data can serve as a reliable proxy for assessing school food consumption.

In general, the IOS method (without combining the SFFQ data) underestimated energy, nutrient, and food group intake estimates by 10–70% compared to the food record method, which is considered the gold standard of dietary assessment. This discrepancy could be attributed to the fact that school cafeterias can only offer limited menu options, allowing university students to complimentarily eat outside the campus to seek other types of dishes. Although the absolute differences were inevitable, the IOS demonstrated comparable participant ranking accuracy to other existing dietary survey methods. Lee et al. [34] assessed the validity of a self-administered, semiquantitative FFQ with 146 items and found that 78% of the participants were categorized into the same or adjacent quartiles for most nutrients. Another method based on a smartphone app indicated that, for food groups, the average percentage of individuals classified in the exact agreement category was 47.1%, while the percentage of individuals classified in the exact agreement or adjacent category was 89.2% [35]. The above validity studies were all referenced against the food diary method.

Spearman’s correlation coefficients between the IOS and the 7DFD for most nutrient and food group intakes were greater than 0.50, which is considered a good outcome according to the criteria used to validate methods for assessing dietary intake [36]. In a study conducted in Finland, the concordance between grocery purchases and food consumption, as measured using an FFQ, ranged from 0.12 to 0.75 based on Gamma coefficients [37]. A study in Chicago reported moderate agreement, with ’ Lin’s concordance correlation coefficient of 0.59, between overall diet quality scores derived from food purchase receipts and 24-hour diet recalls. However, the concordance for specific individual nutrient densities varied widely, ranging from 0.10 to 0.61 [22]. An earlier study in New Zealand revealed acceptable correlations (r_s_>0.30) between household purchases and individual intakes for the percentage of energy from fat, carbohydrate, protein, and sugar but not for sodium or the energy density of beverages [18]. Our study showed stronger correlations, as data from purchased meals better reflect actual food intake than grocery purchase data. This could be due to the consistent and stable food options available in the school canteen. On this basis, a database detailing the nutritional components of menu items was constructed through rigorous weighing and data standardization procedures, which enhanced accurate meal consumption calculations. Furthermore, the Bland–Altman diagrams showed no evidence of systematic bias for energy, protein, fat, or carbohydrate intake. This indicates that IOS data can be used to estimate the distribution of food consumption among individuals in a target group. According to the aforementioned findings, it appears feasible to consider utilizing IOS data for on-campus dietary surveillance to identify segments of the population with potentially inadequate or excessive dietary intake. However, this method is not recommended for comparing the absolute intake to the recommended intake.

The purpose of including the SFFQ was to investigate whether adding it to current IOS data can compensate for the limitation of the canteen menu options. According to our findings, the use of IOS + SFFQ resulted in no significant differences in the consumption of fruits compared to the 7DFD. Liu’s study indicated that our school canteens had an inadequate supply of the above food categories [29]. The SFFQ is an effective solution to address this issue. In addition, the gap between the amounts of energy and necessary nutrients was significantly reduced to less than 10%, which is considered an acceptable outcome in a validity study [36]. However, it was also clearly indicated that the IOS + SFFQ data consistently underestimated the intake of staple foods, meat products, salt, and oil. This finding implies that the SFFQ did not account for other frequently eaten food groups, including packaged snacks, ready-to-eat foods, and late-night snacks, which usually contain high levels of sodium and calories [38, 39]. The association between the IOS + SFFQ and the 7DFD was still somewhat weak (r_s_=0.41) for added sugar, which was not surprising considering how difficult it is to quantify added sugar and the inevitable recall bias. Previous research studies have reported inconsistent agreement (correlation coefficients ranging from 0.25 to 0.50) in validating food assessment methods for measuring sugar or added sugar intake, showing no superior consistency compared to other nutrient intake methods in the same survey [18, 37, 40, 41]. In our research, when completing the 7DFD, detailed information regarding the nutritional ingredients, sweetness levels, and usage of artificial sweeteners in beverages was recorded. However, the SFFQ simply classified added sweetened food intake into limited categories and calculated the sugar content using a rough representative value. Besides, other potential sources of added sugar, such as ice cream, sweet pastries, or cakes, were omitted from the primary study design. These factors may collectively contribute to a lower inter-method correlation. In general, the IOS + SFFQ method shows promise for replacing dietary assessment methods used to obtain precise individual-level data. However, the SFFQ items could be further developed based on the current findings.

Previous studies have shown that statistical analysis of incomplete food shopping data can reveal accurate information on dietary consumption and specific food preferences [42, 43]. Based on our research, a comparison of the regression equations for males and females revealed some interesting findings. Sex differences play a significant role in the nutrient intake and eating habits of university students [12, 44]. In a previous study [29], Liu found that female students had a greater percentage of leftovers in school canteens and were more likely to consume a greater quantity of snacks than male students. In addition, it appears that the IOS data, regardless of whether they were combined with the SFFQ, were a more precise indicator of nutrient intake for males. Specifically, when the IOS data were not combined with the SFFQ, a higher R^2^ suggested a more robust correlation between the estimated and actual observed intakes. For future research, we suggest that when examining the validity of IOS data in various settings or analysing such data, it is vital to pay attention to sex differences between males and females.

As mentioned earlier, there is a lack of studies that have statistically quantified the agreement between automated campus food purchase records and self-reported food intake. Our research lays the groundwork for forthcoming customized and dynamic monitoring and evaluation of dietary intake in school cafeterias. In conjunction with a revamped SFFQ, the IOS can function as a digital tool for health management. It can be used to create electronic records of dietary intake and generate personalized health suggestions. Moreover, our system can provide valuable insights for creating nutrition improvement initiatives for students and staff in the future. Recent evidence also proves that digital health interventions have the capacity to positively influence the dietary habits of specific groups, such as increasing fruit and vegetable consumption [45], increasing overall dietary quality [46], and reducing sodium intake [47]. Additionally, it is worth considering the practical uses of the IOS beyond university campuses, such as in workplaces, communities, and other locations with shared dining areas. In circumstances where data on all three meals are available, the use of the SFFQ is advised to provide a comprehensive understanding of the participants’ overall dietary patterns. In the context of big data, the disparities observed in the IOS + SFFQ compared to the food diary method are considered acceptable, allowing for the retention of absolute food intake values for subsequent dietary assessments. When there is only data available for either lunch or dinner meals, analysing IOS data may not accurately identify those in need of dietary improvement. However, for epidemiological purposes, this type of data is likely sufficient to study the associations between food consumption and health outcomes.

Our study has several strengths. First, the sample included the use of a reliable reference method that helped to minimize measurement errors and systematic biases that may be present in the new method. The food diary, which served as the reference method in our study, is widely regarded as a precise means of capturing dietary intake [13, 30, 48]. Including both workdays and weekends in the recording period of 7 consecutive days allowed for a better picture of the overall diet. Furthermore, previous studies have reported that the validity of the collected information decreased in the latter days of the investigation period [13, 49]. To address these issues, effective quality control measures were implemented, including standardized form-filling instructions, the requirement to upload food pictures, and daily reminders, to minimize missing and invalid data. The study also had notable limitations that need to be acknowledged. The sample included in this study was not a complete representation of all Fudan University students, as the system was initially implemented on the medical school campus. Extensive efforts will be made to expand the database of the remaining canteens at Fudan University. We have also strived to ensure a balanced representation of sex, major, and grade within the medical school, which partially compensated for the lack of a comprehensive sample. While the professional expertise of medical students likely contributed to more precise dietary information and improved the accuracy of our research, this may limit the generalizability of the results to other student populations. Moreover, our study excluded students who ate infrequently at school dining halls to avoid overrepresenting their diet using very little collected data; thus, further research is needed to investigate the relationship between campus food consumption frequency and dietary patterns. Second, importantly, our students were instructed to maintain their usual dietary habits during the study. However, studies have shown that participants may intentionally modify their dietary behaviours to avoid response burden and follow social norms [50] or unintentionally alter them through self-reflection [51, 52], which could confound the current validation results. Fortunately, the use of our IOS method can mitigate this potential observation bias, as food ordering records can be objectively documented. Finally, as mentioned earlier, the SFFQ should be augmented to include common foods not available in school cafeterias, such as packaged snacks and takeaway food.

## Conclusion

Despite the observed disparities in absolute values compared to those of the food diary method, the automatic campus food purchase records obtained from the IOS have the potential to provide a group-level estimation of the daily dietary intake of university students in medical schools located in Shanghai, China. The assessment effectiveness of the IOS data was greater for male students than for female students. The SFFQ can be used as an additional tool to measure the consumption of food groups that may not be readily available in school cafeterias (e.g., fruits, dairy products, and nuts). This helps to improve the accuracy of individual dietary intake evaluations among university students.

### Electronic supplementary material

Below is the link to the electronic supplementary material.

Supplementary Material 1

## Data Availability

The data presented in this study are available upon request from the corresponding author. The data are not publicly available due to the privacy of the study participants.

## Abbreviations

IOS: Intelligent ordering system
7DFD: 7-day food diary
SFFQ: Supplemental food frequency questionnaire
NCDs: Noncommunicable diseases
BMI: Body mass index

## References

[1] Collaborators GBDD (2019). Health effects of dietary risks in 195 countries, 1990–2017: a systematic analysis for the global burden of Disease Study 2017. Lancet.

[2] Zhai FY, Du SF, Wang ZH, Zhang JG, Du WW, Popkin BM (2014). Dynamics of the Chinese diet and the role of urbanicity, 1991–2011. Obes Rev.

[3] Zhou YJ, Du SF, Su C, Zhang B, Wang HJ, Popkin BM (2015). The food retail revolution in China and its association with diet and health. Food Policy.

[4] Fardet A, Aubrun K, Rock E (2021). Nutrition transition and chronic diseases in China (1990–2019): industrially processed and animal calories rather than nutrients and total calories as potential determinants of the health impact. Public Health Nutr.

[5] Arnett JJ (2000). Emerging adulthood - A theory of development from the late teens through the twenties. Am Psychol.

[6] Laska MN, Larson NI, Neumark-Sztainer D, Story M (2012). Does involvement in food preparation track from adolescence to young adulthood and is it associated with better dietary quality? Findings from a 10-year longitudinal study. Public Health Nutr.

[7] Zheng Y, Manson JE, Yuan CZ, Liang MH, Grodstein F, Stampfer MJ (2017). Associations of Weight Gain from Early to Middle Adulthood with Major Health outcomes later in Life. JAMA-J Am Med Assoc.

[8] Tam R, Yassa B, Parker H, O’Connor H, Allman-Farinelli M (2017). University students’ on-campus food purchasing behaviors, preferences, and opinions on food availability. Nutrition.

[9] Pan F, Zhang T, Mao W, Liang D, Luan D, Su C (2022). Eating out of home of urban adults in eighteen provinces (autonomous regions, municipalities) of China in 2017. Wei Sheng Yan jiu = J Hygiene Res.

[10] Sprake EF, Russell JM, Cecil JE, Cooper RJ, Grabowski P, Pourshahidi LK et al. Dietary patterns of university students in the UK: a cross-sectional study. Nutr J. 2018;17. 10.1186/s12937-018-0398-y PMC6172790 30290816

[11] Lupi S, Bagordo F, Stefanati A, Grassi T, Piccinni L, Bergamini M (2015). Assessment of lifestyle and eating habits among undergraduate students in northern Italy. Ann Ist Super Sanita.

[12] Hilger J, Loerbroks A, Diehl K (2017). Eating behaviour of university students in Germany: dietary intake, barriers to healthy eating and changes in eating behaviour since the time of matriculation. Appetite.

[13] Shim JS, Oh K, Kim HC (2014). Dietary assessment methods in epidemiologic studies. Epidemiol Health.

[14] Mezgec S, Eftimov T, Bucher T, Koroušić Seljak B (2019). Mixed deep learning and natural language processing method for fake-food image recognition and standardization to help automated dietary assessment. Public Health Nutr.

[15] Hattab S, Badrasawi M, Anabtawi O, Zidan S (2022). Development and validation of a smartphone image-based app for dietary intake assessment among Palestinian undergraduates. Sci Rep.

[16] Fontana JM, Higgins JA, Schuckers SC, Bellisle F, Pan ZX, Melanson EL (2015). Energy intake estimation from counts of chews and swallows. Appetite.

[17] McClung HL, Ptomey LT, Shook RP, Aggarwal A, Gorczyca AM, Sazonov ES (2018). Dietary Intake and Physical Activity Assessment: current tools, techniques, and technologies for Use in Adult populations. Am J Prev Med.

[18] Eyles H, Jiang Y, Ni Mhurchu C (2010). Use of household supermarket sales data to estimate nutrient intakes: a comparison with repeat 24-hour dietary recalls. J Am Diet Assoc.

[19] Tin ST, Ni Mhurchu C, Bullen C (2007). Supermarket sales data: feasibility and applicability in population food and nutrition monitoring. Nutr Rev.

[20] Clark SD, Shute B, Jenneson V, Rains T, Birkin M, Morris MA. Dietary patterns derived from UK Supermarket Transaction Data with nutrient and socioeconomic profiles. Nutrients. 2021;13(5). 10.3390/nu13051481 PMC8147024 33925712

[21] Dhakal CK, Khadka S (2021). Heterogeneities in consumer Diet Quality and Health outcomes of consumers by Store Choice and Income. Nutrients.

[22] Appelhans BM, French SA, Tangney CC, Powell LM, Wang Y (2017). To what extent do food purchases reflect shoppers’ diet quality and nutrient intake?. Int J Behav Nutr Phys Activity.

[23] Jenneson VL, Pontin F, Greenwood DC, Clarke GP, Morris MA (2022). A systematic review of supermarket automated electronic sales data for population dietary surveillance. Nutr Rev.

[24] Xu SH, Qiao N, Huang JJ, Sun CM, Cui Y, Tian SS et al. Gender Differences in Dietary Patterns and Their Association with the prevalence of metabolic syndrome among Chinese: a cross-sectional study. Nutrients. 2016;8(4). 10.3390/nu8040180 PMC4848649 27023599

[25] de Juras AR, Hsu WC, Cheng YY, Ku LJE, Yu T, Peng CJ (2022). Sex differences in dietary patterns of adults and their associations with the double burden of Malnutrition: a Population-Based National Survey in the Philippines. Nutrients.

[26] Wei L, Fan J, Dong R, Zhang M, Jiang Y, Zhao Q (2023). The Effect of Dietary Pattern on metabolic syndrome in a Suburban Population in Shanghai, China. Nutrients.

[27] Commission (2013). NHaFP. WS/T 428–2013 criteria of weight for adults.

[28] Li SZZ, Mi WQ (2017). Exploration and practice on intelligent ordering system in university. J Cent China Norm Univ Nat Sci.

[29] Liu S, Wang J, He G, Chen B, Jia Y. Evaluation of Dietary Quality based on Intelligent Ordering System and Chinese Healthy Eating Index in College Students from a Medical School in Shanghai, China. Nutrients. 2022;14(5). 10.3390/nu14051012 PMC8912503 35267987

[30] Ortega RM, Perez-Rodrigo C, Lopez-Sobaler AM (2015). Dietary assessment methods: dietary records. Nutr Hosp.

[31] Chinese Nutrition Society. Dietary Guidelines for Chinese Residents. (2022). People’s Medical Publishing House. 2022.

[32] Giavarina D (2015). Understanding bland Altman analysis. Biochem Med.

[33] Pendergast FJ, Ridgers ND, Worsley A, McNaughton SA (2017). Evaluation of a smartphone food diary application using objectively measured energy expenditure. Int J Behav Nutr Phys Act.

[34] Lee Y, Park K (2016). Reproducibility and validity of a semi-quantitative FFQ for trace elements. Br J Nutr.

[35] Béjar LM, Reyes OA, García-Perea MD. Electronic 12-Hour Dietary Recall (e-12HR): comparison of a Mobile phone app for Dietary Intake Assessment with a food frequency questionnaire and four Dietary records. Jmir Mhealth Uhealth. 2018;6(6). 10.2196/10409 PMC6026301 29907555

[36] Lombard MJ, Steyn NP, Charlton KE, Senekal M (2015). Application and interpretation of multiple statistical tests to evaluate validity of dietary intake assessment methods. Nutr J.

[37] Vepsalainen H, Nevalainen J, Kinnunen S, Itkonen ST, Meinila J, Mannisto S (2022). Do we eat what we buy? Relative validity of grocery purchase data as an indicator of food consumption in the LoCard study. Br J Nutr.

[38] Sprake E, Lavin J, Grabowski P, Russell J, Featherstone M, Barker M (2017). Eating habits associated with body weight gain in female university students a UK-based study of Slimming World members. Br Food J.

[39] Wellard-Cole L, Davies A, Chen J, Jung J, Bente KB, Kay J (2021). The Contribution of foods prepared outside the home to the diets of 18- to 30-Year-old australians: the MYMeals Study. Nutrients.

[40] Hamilton S, Mhurchu CN, Priest P (2007). Food and nutrient availability in New Zealand: an analysis of supermarket sales data. Public Health Nutr.

[41] Peralta M, Heskey C, Shavlik D, Knutsen S, Mashchak A, Jaceldo-Siegl K et al. Validity of FFQ Estimates of Total Sugars, added sugars, sucrose and fructose compared to repeated 24-h recalls in Adventist Health Study-2 participants. Nutrients. 2021;13(11). 10.3390/nu13114152 PMC8622229 34836407

[42] Brewster PJ, Guenther PM, Jordan KC, Hurdle JF (2017). The Grocery Purchase Quality Index-2016: an innovative approach to assessing grocery food purchases. J Food Compos Anal.

[43] Taylor A, Wilson F, Hendrie GA, Allman-Farinelli M, Noakes M (2015). Feasibility of a healthy Trolley Index to assess dietary quality of the household food supply. Br J Nutr.

[44] Li KK, Concepcion RY, Lee H, Cardinal BJ, Ebbeck V, Woekel E (2012). An examination of sex differences in relation to the Eating habits and Nutrient intakes of University students. J Nutr Educ Behav.

[45] Alexander GL, McClure JB, Calvi JH, Divine GW, Stopponi MA, Rolnick SJ (2010). A randomized clinical trial evaluating online interventions to improve Fruit and Vegetable Consumption. Am J Public Health.

[46] McCurley JL, Levy DE, Rimm EB, Gelsomin ED, Anderson EM, Sanford JM (2019). Association of Worksite Food Purchases and employees’ overall Dietary Quality and Health. Am J Prev Med.

[47] Dawson J, Campbell KL, Craig JC, Tong A, Teixeira-Pinto A, Brown MA (2021). A text messaging intervention for dietary behaviors for people receiving maintenance hemodialysis: a feasibility study of KIDNEYTEXT. Am J Kidney Dis.

[48] Prentice RL, Mossavar-Rahmani Y, Huang Y, Van Horn L, Beresford SAA, Caan B (2011). Evaluation and Comparison of Food Records, recalls, and Frequencies for Energy and Protein Assessment by using recovery biomarkers. Am J Epidemiol.

[49] Biltoft-Jensen A, Matthiessen J, Rasmussen LB, Fagt S, Groth MV, Hels O (2009). Validation of the Danish 7-day pre-coded food diary among adults: energy intake v. energy expenditure and recording length. Br J Nutr.

[50] Tang JS, Haslam RL, Ashton LM, Fenton S, Collins CE (2022). Gender differences in social desirability and approval biases, and associations with diet quality in young adults. Appetite.

[51] Kristjansdottir AG, Andersen LF, Haraldsdottir J, de Almeida MDV, Thorsdottir I (2006). Validity of a questionnaire to assess fruit and vegetable intake in adults. Eur J Clin Nutr.

[52] Lividini K, Fiedler JL, Bermudez OI (2013). Policy implications of using a Household Consumption and expenditures Survey versus an observed-weighed Food Record Survey to Design a Food Fortification Program. FoodNutr Bull.

